# Educational Attainment as a Super Determinant of Diet Quality and Dietary Inequities^☆^

**DOI:** 10.1016/j.advnut.2025.100482

**Published:** 2025-07-17

**Authors:** Dana Lee Olstad, Lynn McIntyre

**Affiliations:** Department of Community Health Sciences, Cumming School of Medicine, University of Calgary, Calgary, Alberta, Canada

**Keywords:** diet quality, socioeconomic inequities, dietary inequities, health inequities, educational attainment, income, gaps, gradients, socioeconomic position

## Abstract

Inequities in diet quality are evident worldwide and reflect structural disadvantages. There is increasing evidence that dietary inequities may be most meaningful in relation to educational attainment, a finding that contradicts the common belief that dietary inequities are primarily attributable to material disadvantage (i.e. inadequate incomes). Moreover, diet quality declines with each step down the educational ladder, and therefore, these educational inequities affect all of society. The purpose of this perspective is to posit that educational attainment is a key structural stratifier of diet quality and dietary inequities—what we term a super determinant—and that greater research attention should be given to interrogating pathways through which educational attainment shapes diet quality. To inform our perspective, we conducted extensive keyword searches in PubMed and Google Scholar to identify concepts, theories, and empirical data pertaining to educational inequities in diet quality, health, and mortality, followed by a conceptual synthesis of findings. On the basis of these findings, we first describe pathways through which educational attainment shapes diet quality. We then demonstrate that educational inequities in diet quality are often much larger than they are for income. For instance, absolute gaps and gradients in Healthy Eating Index-2015 scores between the most and least educated adults were 7–11 points in Canada, whereas they were just 2–5 points in relation to household income. We provide converging evidence related to large and growing educational inequities in diet quality, health, and mortality internationally. We subsequently consider an important counterfactual—that the affordability of a healthy diet is the key determinant of dietary inequities—and empirically demonstrate that economic factors are not primary drivers of socioeconomic inequities in diet quality. We conclude that attributing dietary inequities primarily to the higher costs of healthy foods is overly simplistic and ignores the critical role of educational attainment as a structural stratifier of dietary inequities.

**Statement of significance**

We propose that educational attainment is a super determinant of diet quality and dietary inequities. Although many within the nutrition community maintain that dietary inequities are primarily a consequence of inadequate income relative to the higher costs of healthier foods, much evidence reveals the critical role of educational attainment in driving dietary inequities. By exploring the evidence that shows that educational attainment is a unique and powerful determinant of diet quality and dietary inequities, new topics of scientific inquiry directed at improving diet quality and reducing dietary inequities can be used to promote population health and healthy eating.

## Introduction

**FIGURE 1:**
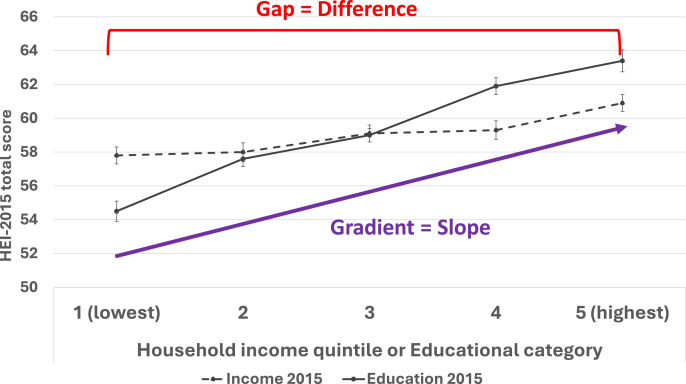
Absolute gaps and gradients in Healthy Eating Index-2015 (HEI-2015) scores in Canada in 2015 in relation to household income quintiles and educational attainment. Educational categories: less than high school; high school; some postsecondary; Bachelor’s degree; higher than Bachelor's degree.

Public health nutrition is deeply concerned with the nutritional health of the population and with differences in diet quality between population subgroups. When these between-group differences in diet quality are due to unfair or modifiable social and structural factors, such as income or educational attainment, the result is dietary inequity [1]. Although gaps in diet quality refer to differences in diet quality between those at the extreme ends of the socioeconomic spectrum, gradients refer to systematic variations in diet quality across the full spectrum of disadvantage (Figure 1) [2].

Dietary inequities persist and are increasing in some cases [[3], [4], [5], [6]]. This concerns the nutrition community because dietary inequities may contribute to health inequities [7]. We begin this perspective by asking: are socioeconomic inequities in diet quality important? Our answer: it depends on the indicator in question and whether one believes the size (i.e. the gap) or the slope (i.e. the gradient) of the inequity is most important.

Importantly, as we show below, inequities in diet quality are evident worldwide and reflect structural disadvantage within and between populations. Yet, the small size of some of these differences suggests they may not matter in practice, as they are unlikely to yield large inequities in health outcomes. Moreover, a minority of people adhere to dietary recommendations, regardless of their socioeconomic position (SEP) [[3], [4], [5], [6]]. Poor diet quality is therefore a generalized phenomenon across the socioeconomic spectrum. This suggests that equalizing diet quality across the socioeconomic spectrum may do little to improve population health or to reduce health inequities. However, as we go on to describe, there is increasing evidence that dietary inequities may be more meaningful in relation to educational attainment, a finding that contradicts the commonly held belief that dietary inequities are primarily attributable to the material disadvantages imposed by inadequate incomes.

The purpose of this perspective is to posit that educational attainment should be regarded as a key structural stratifier of diet quality and dietary inequities—what we term a super determinant—and that greater research attention should be given to interrogating pathways through which educational attainment shapes diet quality. To support our perspective, we first describe the place of educational attainment within indicators of SEP. We then explore educational gaps and gradients in diet quality internationally and in Canada, and how these dietary trends mirror those for other health-related outcomes. We subsequently consider an important counterfactual—that the affordability of a healthy diet is the key determinant of dietary inequities—and conclude by summarizing the implications of our perspective for practice, policy, and research.

## Methods

We conducted a selective review of the literature to identify key concepts, theory, and empirical data pertaining to educational inequities in diet quality, health, and mortality, followed by a conceptual synthesis of findings. To do so, we executed extensive searches in PubMed and Google Scholar using keywords related to education, income, inequities, diet, nutrition, health, and mortality. The selection of articles for full-text review was based on article titles and abstracts. Relevant articles were read in full, and findings were summarized. Relevant citations from each article were also retrieved, read, and summarized. Together, we interrogated the core evidence and pertinent documentation for our perspective argumentation. In total, evidence from the most relevant 86 articles was used to inform our perspective, as cited throughout this manuscript.

### Socioeconomic position and diet quality

It is widely accepted, and empirically observed, that many individuals with lower incomes cannot afford the higher costs of a healthy diet consisting of whole, nutrient-dense foods, and therefore default to purchasing their highly processed and nutrient-poor counterparts [8,9]. Alongside income, the nutrition literature also considers educational attainment and several other indicators of SEP as determinants of diet quality.

SEP is a complex, multidimensional construct that represents individuals’ social and economic position in relation to others. SEP is both defined and generated by social structures and processes that allocate power and influence according to factors such as income, educational attainment, and occupation [1]. Each indicator of SEP captures distinct, yet also overlapping, axes of social stratification that shape access to health-promoting resources and exposure to adverse environmental conditions, and their relevance differs across the life course [1,[10], [11], [12]]. Thus, there is no single “best indicator” of SEP that fully encompasses the array of factors that shape individuals’ dietary patterns and health at all life stages.

Household income is an indicator of SEP that reflects access to the material resources required to support healthy dietary patterns, such as having sufficient income to afford nutritious foods, transportation to procure food, cooking facilities to prepare food, and a secure place to consume meals. Accordingly, socioeconomic inequities in diet quality in relation to household income are usually assumed to indicate that money, and the material resources and services that money can buy, are driving these inequitable dietary patterns [4].

Given its specificity, household income is an important indicator of SEP when material mechanisms are of interest. However, income can also fluctuate markedly, is difficult to recall accurately, and usually has substantial missing data [10]. Moreover, the adequacy of household income can be unclear when values are not adjusted for household size, assets, debts, and local costs of living, and the distribution of income within a household may not always be egalitarian, such as when 1 partner exerts more control over a household’s finances. Subjective measures of household income also exist, which can help to overcome some of these limitations, including perceived income adequacy [[13], [14], [15]] and household food insecurity [16,17].

In contrast, educational attainment is a much broader indicator of SEP that encompasses material pathways that shape diet quality (i.e. in part because educational attainment is a key determinant of income), along with many others such as individuals’ occupations and working conditions (e.g. shift work that leads to irregular meal times [18], job strain that promotes stress-related eating), social supports and networks (e.g. peers who eat healthfully), cultural capital [19] (e.g. frequenting high-end restaurants, cooking with organic ingredients), health-related knowledge and skills (e.g. nutrition literacy), and present- compared with future-orientation [[20], [21], [22], [23]] (e.g. focus on immediate hedonic rewards compared with future health goals) [24,25]. Given that educational attainment is such a broad indicator, when gradients in diet quality in relation to educational attainment are observed, the relevant mechanisms are often unclear, although knowledge-related mechanisms are commonly invoked [26,27].

Educational attainment—whether measured based on the number of completed years or earned credentials—has many advantages as an indicator of SEP. First, formal education is typically completed early in life and tends to remain stable over time. As such, it better reflects lifetime exposures, inherently incorporates a lag time for effects to materialize, and is less likely to be affected by reverse causality [28]. Second, educational attainment is a comprehensive indicator that reflects a variety of cultural, material, social, and cognitive assets, and thus can be used to examine multiple pathways through which SEP shapes dietary intake [24,25]. Third, educational attainment has strong measurement properties because it is easy to recall and classify, and typically has little missing data [28]. Education is also distinct from other social resources because it is embodied within individuals and is a source of human capital [24,29]. Although income can decline or a job can be lost, once individuals learn new information and develop cognitive and noncognitive capabilities related to reasoning, problem solving, decision making, and dependability, this human capital cannot be taken away and positively shapes individuals’ health-related practices and outcomes across the life course [24,29,30].

Nevertheless, educational attainment also has limitations as an indicator as it only captures formal education and does not reflect the quality of education received [28]. Its relevance to the health of younger adults who have not yet completed their education and to older adults who have retired is also unclear [31]. Yet, despite its limitations, educational attainment is perhaps one of the most useful indicators of SEP given its comprehensiveness, stability, temporal precedence, and strong measurement properties.

Although the higher costs of many healthier foods in relation to income—and the affordability of basic necessities more generally—undoubtedly contribute to socioeconomic inequities in diet quality [9], limited attention has been given to the role of educational attainment in shaping socioeconomic inequities in diet quality in the nutrition literature. This is a critical oversight given its many strengths as an indicator of SEP, and its strong independent associations with diet quality [26,32,33]. Indeed, as we show in the following sections, educational attainment is perhaps the most fundamental determinant of diet quality and dietary inequities.

### Socioeconomic inequities in diet quality in Canada

Gaps and gradients in diet quality at a population level can be measured along a socioeconomic gradient, allowing them to be properly classified as dietary inequities. We investigated trends in socioeconomic inequities in diet quality between 2004 and 2015 among large, nationally representative samples of adults and children in Canada [4,34]. Dietary inequities were quantified using absolute and relative gaps (between the most and least disadvantaged) and absolute and relative gradients (Slope Index of Inequality and Relative Index of Inequality, respectively) for multiple disadvantaged groups. Absolute gaps and gradients in diet quality [based on the 100-point Healthy Eating Index-2015 (HEI-2015)] between the most and least educated adults were 7–11 points, whether educational attainment was measured at the individual or household level. In contrast, gaps and gradients in diet quality were smaller in relation to household income—at just 2–5 points. Figure 1 illustrates these differences in the total population in 2015. Similar discrepancies in the size of dietary gaps and gradients in relation to household educational attainment and income were evident among children [34]. Moreover, trend analyses revealed that the diet quality of all adults improved over time—except among those without a high school diploma—and gaps and/or gradients in diet quality by individual/household educational attainment widened among adult males and 6–11 y old children.

The literature suggests that 5 points is a clinically meaningful difference in HEI-2015 scores [35]—a threshold surpassed by the educational but not the income-related dietary gaps and gradients we observed. We also found that dietary gradients for both income and education were somewhat larger than their corresponding dietary gaps, with the educational gradients being particularly large. This finding indicates that educational inequities in diet quality do not merely afflict those with the lowest attainment. Rather, diet quality declines with each step down the educational ladder, and therefore educational inequities transect all tiers of society [2].

Three subsequent studies confirmed the primacy of educational attainment as a structural stratifier of dietary inequities in Canada. An analysis of trends between 2004 and 2015 showed that the most consistent gaps and gradients in the proportion of energy consumed from ultraprocessed (i.e. industrially manufactured products that contain little or no whole foods [36]) and minimally processed foods were for household and individual educational attainment [37]. Adults with lower educational attainment consumed more ultraprocessed and fewer minimally processed foods than those with higher educational attainment. Notably, there were no inequities in the intake of ultraprocessed foods in relation to household income. Moreover, gaps and gradients in intake of minimally processed foods were in the opposite direction from what was expected—those with the lowest incomes consumed more minimally processed foods than those with the highest incomes.

In a second study of dietary intake in 2015, we identified SEP intersections that best predicted the diet quality (HEI-2015 scores) of a nationally representative sample of adults in Canada (Doan et al, manuscript submitted for publication). The top 3 SEP intersections identified by conditional random forests were educational attainment and Indigenous identity and race/ethnicity; educational attainment and household food insecurity; and educational attainment and sex/gender. Moreover, of the 12 SEP indicators included in our models, the variable that improved predictions of diet quality to the greatest extent was educational attainment, with a variable importance measure of 14.1. By comparison, the variable importance measure for household income was just 1.1.

Finally, in a cross-country comparative study, we also found that associations between educational attainment and diet quality (HEI-2020 scores) were stronger than associations between perceived income adequacy and diet quality, and between household food insecurity (a marker of material deprivation) and diet quality in both Canada and the United States (Doan et al, manuscript submitted for publication). Associations between educational attainment and diet quality were only slightly attenuated when adjusted for perceived income adequacy and household food insecurity, indicating that economic factors did not mediate a substantial proportion of associations between educational attainment and diet quality in either country.

### Socioeconomic inequities in diet quality internationally

Evidence confirms that educational gaps in diet quality are also a global concern, although their size varies considerably among countries [5,6]. Such cross-country differences in inequities in diet quality are not unexpected due to underlying differences in their populations and socioeconomic and political contexts. One cross-national analysis of 185 countries confirmed that individuals with higher educational attainment had better diet quality than those with lower educational attainment, with average gaps of ∼2–3 points based on the 100-point alternate HEI [6]. Gaps were larger in Latin American/Caribbean and South Asian countries (∼4–5 points) than in other world regions (∼1–2 points) [6]. In another study, individuals with lower educational attainment living in 12 European nations tended to have poorer nutritional intakes than those with higher educational attainment [5].

Educational gaps in diet quality are also evident in studies of individual countries. For instance, in the United States, diet quality is also patterned by education as it is in Canada. However, the 2-point difference in HEI-2015 scores between the most and least educated adults in the United States in 2015 [3] was much smaller than the average 9-point gap between educational groups that we found in Canada that same year, even after accounting for measurement differences [4]. Also, in contrast to Canada, dietary gaps in relation to household income and educational attainment appear to be relatively similar in size in both the United States [3,38,39] and the United Kingdom [40]. There is also mixed evidence relating to trends in dietary inequities in the United States, with some studies finding widening gaps according to household income alone [39], educational attainment alone [41], both [3] or neither [38] between 1999 and 2016. Nonetheless, inequities in diet quality in several other countries do resemble those in Canada. For instance, in Australia and the Netherlands, there is a clearer patterning of diet quality in relation to educational attainment than there is in relation to household income [42,43], and dietary inequities in relation to educational attainment are large in the Netherlands [43,44].

Beyond findings pertaining to dietary gaps between educational groups, a growing body of observational studies point to the importance of educational attainment as a more important determinant of diet quality than income. For instance, large-scale data from >1.16M United States adults [45], a systematic review of 40 international studies [46] and a survey of 1092 adults in the Canadian province of Quebec [26] found that educational attainment was more strongly associated with diet quality than income. Similar observations have been made in the Netherlands [43,47] and Norway [48]. Another United States study found that changes in educational attainment were more robust predictors of changes in diet quality than changes in income [32].

This convergence of findings from multiple studies suggests that inequities in diet quality are structured by educational attainment in Canada and throughout the Western world, and that some of these inequities may be widening. This challenges the common assumption that socioeconomic inequities in diet quality are primarily attributable to the higher costs of healthier foods. Although economic factors undoubtedly contribute to dietary inequities, these inequities must extend beyond the material disadvantages imposed by inadequate incomes to encompass differentials in power, status, knowledge, social networks, and other factors that are captured by educational attainment. We next investigate if educational attainment as a super determinant plays a unique and powerful role in other health outcomes. If so, there may be deeper structural forces at play.

### Socioeconomic inequities in morbidity and mortality internationally

The aforementioned body of data pertaining to inequities in diet quality is supported by converging evidence related to large and growing educational inequities in health and mortality in Canada, the United States, and Europe over the past several decades [30,[49], [50], [51], [52], [53], [54], [55]]. Indeed, completion of postsecondary education—and a Bachelor’s degree in particular—has been shown to structure a host of outcomes related to physical activity, smoking, sleep, pain, disability, chronic disease, and mortality [30,[49], [50], [51], [52], [53], [54],[56], [57], [58], [59]], including deaths of despair associated with drugs, alcohol, and suicide [[60], [61], [62], [63]]. The size of these inequities is considerable, as the number of deaths attributable to low education in the United States exceeds deaths attributable to poverty, income inequality, racial segregation, and low social support [64]. With each additional year of education, individuals increasingly display a host of positive health-related outcomes, including better diet quality, improved mental health, longer sleep, lower incidence of chronic disease, and greater longevity [30,[49], [50], [51], [52], [53], [54],[58], [59], [60]]. Educational attainment is also a better predictor of life expectancy than race/ethnicity [50] and a better predictor of physiologic health markers across the life span than wealth and household income [65]. It is estimated that each additional year of education increases longevity by 4.8 mo [58].

Specific to the Canadian context, whereas absolute risk differences in premature mortality by household income narrowed among men between 1991 and 2016, they increased in relation to educational attainment [51]. Among women, risk differences in relation to both income and educational attainment widened; however, the degree of widening was much larger in relation to educational attainment. In addition, premature mortality rates have declined over time among all socioeconomic groups except among women without a high school diploma in both Canada [51] and the United States [55], a finding that mirrors our own findings with respect to trends in diet quality [4].

Overall, we have shown that educational inequities in diet quality in Canada and internationally clearly comport with global patterns for a variety of dietary and health-related outcomes. The remarkable consistency with which educational attainment drives inequities in diet quality and health is worth further investigation, particularly at a time when dietary and health inequities are rising. We are not the first to posit that educational attainment is a super determinant of health-related outcomes. Others have suggested that educational attainment constitutes an overarching and enduring fundamental social determinant of health that shapes multiple health-related outcomes in diverse contexts [24]. Before fixating on educational attainment as a super determinant of diet quality and dietary inequities, it behooves us to consider an important counterfactual—that the affordability of a healthy diet is the key determinant of dietary inequities.

### The affordability of healthy dietary patterns

Despite the common assertion that socioeconomic inequities in diet quality are primarily a function of the higher costs of healthy foods in relation to household income, the evidence in this respect is actually quite mixed. It is notable that mean diet quality is suboptimal in all countries [6], despite marked differences in the affordability of healthy diets among them. For instance, healthier diets tend to be more costly than less healthy diets in the Netherlands [66], Canada [67], and the United States [8,[68], [69], [70]], but this is not the case in Australia [71,72] or Mexico [73]. Moreover, even in countries where healthier diets are costlier than unhealthy diets, the difference in costs is often very small. For instance, in Canada, healthier diets cost on average Canadian dollar (CAD) $1.09–$1.39 more per day relative to less healthy diets [67,74]. This amounts to just $1.06–$1.45 more per day in 2025 US Dollar (USD). In the United Kingdom, dietary patterns that are more consistent with dietary recommendations cost 3%–17% more than those that do not meet recommendations [75,76]. Similarly, greater adherence to a Mediterranean diet has no impact on daily dietary cost in Canada [77] and increases food expenditures by just 5.4% in the United Kingdom [76]. Conclusions from these individual studies are supported by a meta-analysis which found that healthier dietary patterns cost just USD $1.54 more per day than less healthy patterns [78] or $2.30 more per day in 2025 dollars. Overall, in North America, a nutritionally adequate diet for a healthy adult female can be procured for just USD $2.82/d when adjusted to 2025 dollars [79]. Globally, the cost to purchase a healthy diet for an active 30-year old female (based on 2,330 kcal/d) was estimated to be $3.68/day in 2021 purchasing power parity dollars [80].

Evidence also indicates that increasing food expenditures produces only marginal gains in diet quality, highlighting that the amount of money spent on food is not as tightly linked to the healthfulness of dietary patterns as is commonly thought. For example, in the United States, increasing food expenditures by 20% only improves diet quality by 1.9%—a very small improvement for a substantial change in cost [81]. In contrast, switching from purchasing food in restaurants to shopping in grocery stores yields greater gains in diet quality while simultaneously reducing food expenditures. In addition, sociodemographic factors, including educational attainment, smoking, gender, age, and race/ethnicity are much stronger predictors of diet quality than food expenditures [81].

Taken together, these data underscore the reality that even in countries where healthier diets tend to be more costly than unhealthy diets, the differences in cost tend to be small, and a healthy diet can be procured with relatively modest financial outlay.

### The affordability of healthy dietary patterns by SEP

In addition to understanding the price of healthy eating more generally, it is important to understand the affordability of healthier dietary patterns by SEP. One study in the Netherlands found that healthy diets were equally affordable for all educational groups, costing ∼20% of household income [66]. Mediation analyses can provide more direct evidence in this respect by showing the extent to which the higher prices of healthier foods can explain dietary inequities. However, the evidence in this respect is limited and mixed. Two studies in the Netherlands found that food costs/budgets did not mediate associations between educational attainment and diet quality [43] or mediated a negligible proportion of them (2%–7%) [44]. By contrast, in the United Kingdom and the United States where incomes are more unequal, food costs mediated ≤53% (based on occupation) and 76% (based on educational attainment) of dietary inequities, respectively [9,82]. However, another United States study found that food cost mediated <10% of dietary inequities according to both educational attainment and income [83]. Notably, these studies are quite dated, and thus, additional mediation analyses are essential, particularly in the post-COVID era.

One reason why food costs may not mediate a substantial proportion of dietary inequities could be because food prices do not factor prominently into the food purchasing decisions of individuals with a lower SEP. Interestingly, there is evidence to support this hypothesis as several studies have shown that individuals with lower incomes and educational attainment are less sensitive to food prices than those with higher incomes and educational attainment [84,85]; however, this is not a universal finding [86]. In some cases, individuals with lower incomes have been shown to be relatively insensitive to the cost of food [85,87] and capable of achieving a similar diet quality as those with much higher incomes when they prioritize nutritional concerns [86]. In 1 novel study that equalized the available food budgets of women with high and low incomes, the food purchases of women with low incomes were still nutritionally inferior relative to those of women with higher incomes, indicating that costs were not the key barrier in this group of women [88]. These collective findings lend further credence to the notion that food costs are not the primary determinant of dietary inequities and that other factors must be considered.

The primacy of educational attainment over income is also seen in studies where the protective effects of income on health diminish with higher levels of income, whereas additional education continues to yield health dividends even at higher levels of attainment [89,90]. We similarly found that the diet quality of adults in Canada with more than a Bachelor’s degree was higher than the diet quality of individuals in the highest income quintile, suggesting that the benefits of income on diet quality plateau at lower levels than they do for educational attainment [4]. The marginal dietary benefits that accrue to increasing incomes can also be seen in data from the United States indicating that increasing mean income from $45,744 to $55,744 (22%) raises HEI-2005 scores by just 0.14 points (out of 100) [81] and that differences in food expenditures account for < 5% of differences between the diet quality of households above and below the poverty line, whereas differences in educational attainment account for 17%–45% of the gap [91]. Mediation analyses have also shown that economic resources account for < 20% of associations between educational attainment and health-related practices collectively, including dietary intake, physical activity, smoking, alcohol intake, and preventive care [20]. In another study, household income in adulthood did not mediate associations between educational trajectories in early adulthood and diet quality at age 46 [92], whereas in another, income mediated just 6%–9% of associations between educational attainment and diet quality [93]. Finally, several randomized controlled trials have found that providing additional funds to individuals with low incomes—even as much as $1000/mo for 3 y—reduces food insecurity but has little to no effect on diet quality [[94], [95], [96], [97], [98]].

Taken together, this body of literature further underscores the reality that economic factors are not crucial drivers of socioeconomic inequities in diet quality in general, nor are they the primary drivers of educational inequities in diet quality. Moreover, it appears that once basic needs are met, the impact of additional income on health and health-related practices may be limited, whereas each additional year of education continues to provide health-related benefits.

### Integration of evidence and the way forward

Overall, our findings pertaining to large and growing educational inequities in diet quality, health, and mortality in Canada and elsewhere merit further investigation as they indicate that these inequities may be related to deep-seated structural disadvantages. We have also shown that food costs play a more modest role in explaining dietary inequities than is commonly posited and that economic factors are not the primary drivers of educational inequities in diet quality. Regardless of income and educational attainment, diet quality is universally poor across the socioeconomic spectrum in all countries [6]—leading us to question whether equalizing diet quality across the socioeconomic spectrum will do much to reduce health inequities. There is little reason to expect that increasing the diet quality of those most disadvantaged in society to a somewhat higher—yet still poor—diet quality, will have much impact. Evidence of widening dietary inequities should raise concern, however, as large changes could lead to adverse population health consequences, particularly if the widening occurs via reductions in diet quality at the lower end of the socioeconomic spectrum or if such changes are a marker of increases in structural drivers of health harm for populations lacking educational equity.

Overall, the evidence presented herein suggests that educational attainment is a key determinant of diet quality that should be accorded greater research attention. It is also critical to investigate how educational attainment interacts with other dimensions of SEP to confer dietary risk differently for more precisely defined population subgroups within a precision public health framework [99]. Future studies should continue to follow trends in educational inequities in diet quality over time in Canada, the United States, and other nations, including at subnational levels (e.g. provinces, states) and for subgroups of the population (e.g. racial/ethnic subgroups, Indigenous people, immigrants, the unemployed). Greater attention to the heterogeneity of social positions can help to identify the upstream origins of dietary inequities and thereby identify more specific points of intervention to reduce them [99].

Our findings of large and growing educational inequities in diet quality and the lack of improvement in diet quality among those in the least educated group over time challenge the common assertion that a “rising tide” of educational attainment will “lift all boats.” Our analyses using nationally representative data indicate that at least 10% of adults in Canada are being left behind. These trends mirror those for other health-related outcomes internationally, suggesting that Canada is unlikely to be an outlier in this respect.

If we wish to reduce dietary inequities, we must first understand the relevant mechanisms at play. Nutrition scientists often search for determinants of dietary patterns among exposures that can be readily quantified; however, doing so may miss the actual causative mechanisms. Indeed, we know relatively little about which mechanisms are primarily responsible for educational gradients in diet quality and other health-related outcomes due to the overwhelming use of educational attainment as an exposure variable [100]. Uncovering these mechanisms will require more mediation analyses and measuring education in more creative ways beyond simple attainment of a credential, such as by considering the type, timing, and quality of education received [28].

Finally, a clear implication of our findings is that inequities in diet quality are not inevitable and are partly attributable to policy choices that have shaped educational attainment over the past several decades. As such, policies that increase educational attainment could be accorded a higher priority. Such policies might include those that remove barriers to acquiring higher education such as student bursaries and loans, reducing tuition fees, childcare subsidies, educational leaves from employment, among others. Nevertheless, it is important to bear in mind that policies that facilitate the achievement of higher education will change the distribution of educational attainment at a population level, which could lead to the unintended negative consequence of further marginalizing individuals with lower attainment [101]. Studies that directly investigate mechanisms through which educational attainment shapes diet quality may therefore better suggest possible interventions.

### Limitations

The size of socioeconomic inequities will always be sensitive to the SEP classifications that are used. Our aforementioned analyses of trends in Canada used 5 categories to characterize both household income and educational attainment. These categories reflect commonly used and meaningful thresholds in relation to poverty (e.g. the lowest income quintile was below the low-income cut-off used to denote poverty in Canada) and completion of key educational milestones (e.g. the lowest education group had not completed a high school degree). Each group also encompassed meaningful proportions of the population, and we used survey weights to ensure our analyses were nationally representative. Sensitivity analyses using alternative classifications did not change our conclusions. Moreover, we characterized inequities using both dietary gaps and gradients, and the results were very similar between the 2 measures. Although dietary gaps represent differences between the extreme ends of the socioeconomic spectrum, dietary gradients consider the slope of the entire distribution and the size of each group and are therefore less sensitive to classification thresholds. The veracity of these findings was also supported by 3 subsequent studies, as previously described. As we are the first and only team to have comprehensively characterized absolute and relative dietary gaps and gradients using multiple indicators of SEP in nationally representative samples, we recommend that others conduct similar analyses to better characterize dietary inequities internationally. Finally, dietary misreporting is always a concern with self-reported dietary intake data. However, our analyses were based on data from 24-h dietary recalls, which are the most robust means of dietary assessment [102]. Moreover, the odds of misreporting did not differ by SEP in our analyses.

In conclusion, in returning to our original question—are inequities in diet quality important—our answer remains unchanged. It depends on the indicator (i.e. income or education) in question and whether one believes the size or the slope of the inequity is most important. Although diet quality is patterned by household income in Canada and many other nations, the gaps are small and the gradients shallow. Educational attainment, however, appears to be more important in structuring dietary inequities in several countries—exhibiting larger gaps and steeper gradients—both of which surpass clinically meaningful thresholds. Reducing these educational gradients in diet quality will be an especially formidable challenge as it will require interventions that improve diet quality at a faster rate among the least relative to the most educated [2].

Attributing dietary inequities primarily to the higher costs of healthy relative to less healthy foods greatly oversimplifies the nature of dietary inequities and ignores the critical role of educational attainment as a structural stratifier of dietary inequities. We have documented the case for considering educational attainment as a super determinant of diet quality and dietary inequities. Large educational inequities in diet quality merit concern and further investigation to understand the underlying mechanisms. However, the available data on the size and scope of educational inequities in diet quality internationally remain limited. To better understand global patterns in dietary inequities, researchers in other countries should comprehensively characterize absolute and relative dietary gaps and gradients using multiple indicators of SEP in nationally representative samples. A better understanding of these cross-national variations can help to understand potential mechanisms at play and policy solutions. In future papers, we will explore the current state of the evidence pertaining to potential mechanisms underlying educational gaps and gradients in diet quality and whether they make a meaningful contribution to health inequities, consider implications for policy and practice, and outline a research agenda for the public health nutrition community to tackle educational inequities in diet quality.

## Author contributions

Both authors read and approved the final manuscript.

## Data availability

All data sources used in preparing this manuscript have been cited in the text and thus no additional provisions have been made for data sharing.

## Funding

The authors reported no funding received for this study.

## Conflict of interest

The authors report no conflicts of interest.
